# Transposition of the *Tourist*-MITE *mPing *in yeast: an assay that retains key features of catalysis by the class 2 *PIF/Harbinger *superfamily

**DOI:** 10.1186/1759-8753-1-5

**Published:** 2010-02-01

**Authors:** C Nathan Hancock, Feng Zhang, Susan R Wessler

**Affiliations:** 1 Plant Biology Department, University of Georgia, Athens, GA 30602, USA; 2 Department of Genetics, Cell Biology and Development, University of Minnesota, Minneapolis, MN 55455, USA

## Abstract

**Background:**

*PIF/Harbinger *is the most recently discovered DNA transposon superfamily and is now known to populate genomes from fungi to plants to animals. Mobilization of superfamily members requires two separate element-encoded proteins (ORF1 and TPase). Members of this superfamily also mobilize *Tourist*-like miniature inverted repeat transposable elements (MITEs), which are the most abundant transposable elements associated with the genes of plants, especially the cereal grasses. The phylogenetic analysis of many plant genomes indicates that MITEs can amplify rapidly from one or a few elements to hundreds or thousands.

The most active DNA transposon identified to date in plants or animals is *mPing*, a rice *Tourist*-like MITE that is a deletion derivative of the autonomous *Ping *element. *Ping *and the closely related *Pong *are the only known naturally active *PIF/Harbinger *elements. Some rice strains accumulate ~40 new *mPing *insertions per plant per generation. In this study we report the development of a yeast transposition assay as a first step in deciphering the mechanism underlying the amplification of *Tourist*-MITEs.

**Results:**

The ORF1 and TPase proteins encoded by *Ping *and *Pong *have been shown to mobilize *mPing *in rice and in transgenic *Arabidopsis*. Initial tests of the native proteins in a yeast assay resulted in very low transposition. Significantly higher activities were obtained by mutation of a putative nuclear export signal (NES) in the TPase that increased the amount of TPase in the nucleus. When introduced into *Arabidopsis*, the NES mutant protein also catalyzed higher frequencies of *mPing *excision from the *gfp *reporter gene. Our yeast assay retains key features of excision and insertion of *mPing *including precise excision, extended insertion sequence preference, and a requirement for two proteins that can come from either *Ping *or *Pong *or both elements.

**Conclusions:**

The yeast transposition assay provides a robust platform for analysis of the mechanism underlying transposition catalyzed by the two proteins of *PIF/Harbinger *elements. It recapitulates all of the features of excision and reinsertion of *mPing *as seen in plant systems. Furthermore, a mutation of a putative NES in the TPase increased transposition both in yeast and plants.

## Background

Class 2 DNA transposons were discovered in maize over 60 years ago with the genetic characterization of the *Ac*/*Ds *family of autonomous and nonautonomous elements by McClintock [1]. Since then, DNA transposons have been found in all kingdoms of life and have been characterized into at least 10 superfamilies, based on the sequence of the element-encoded transposase protein [2]. The newest superfamily is *PIF*/*Harbinger*, whose existence only came to light in the last decade. *PIF*/*Harbinger *derives its name from the two founding elements: *Harbinger *from *Arabidopsis thaliana *and *PIF *from *Zea mays*, discovered by computational and genetic analyses, respectively [3,4].

Several features of transposition distinguish *PIF*/*Harbinger *from the other superfamilies. First, virtually all coding elements characterized to date contain two genes, ORF1 and TPase [5,6]. Unlike *CACTA *elements where alternative splicing produces multiple proteins [7,8], the two genes of *PIF*/*Harbinger *elements appear to be independent [5,6]. Both the ORF1 and TPase proteins are required for transposition [9,10]. Second, where analysed, excision is usually perfect as both the element and one copy of the 3 bp target site duplication (TSD) generated upon insertion is excised from the donor site [9,10]. This differs from all previously characterized plant transposable elements where the majority of excision events leave a footprint or deletion at the excision site [11]. Third, *PIF*/*Harbinger *elements display an extended target sequence preference: 9 bp in plants [4,9,12] and 15 bp in vertebrates [6,10].

Another distinguishing feature of this superfamily is that *PIF/Harbinger *elements are responsible for the generation and amplification of *Tourist-like *miniature inverted repeat transposable elements (MITEs), one of the two predominant MITE families (the other being *Stowaway*). MITEs are small (100-500 bp) non-coding elements with the ability to amplify rapidly from one or a few near-identical elements to hundreds or thousands of copies [13]. MITEs comprise ~5% of the rice genome [14] and are abundant in the genomes of some animals including mosquitoes [15], zebrafish [16] and humans [17,18]. Where MITEs have been analysed on a genome-wide basis, they appear to play a significant role in gene evolution as they are abundant and insert preferentially into or near genes [19]. In order to understand the success of *Tourist*-MITEs we need to first understand the transposition mechanism of *PIF*/*Harbinger *elements. With this goal in mind, we focus in this study on the rice *mPing *element, which is the only known active MITE.

Computational analysis of the sequenced genome of the rice (*Oryza sativa*, *japonica*) cultivar Nipponbare led to the identification of *mPing *[20]. *mPing *was independently discovered to be actively transposing in the rice strain Gimbozu/EG4 [21] and in rice anther culture [22]. Further analysis revealed that this 431 bp *Tourist-like *MITE is a perfect deletion derivative of the 4.5 kb *Ping *element, which is present as a single copy in the Nipponbare genome and is a member of the *PIF*/*Harbinger *superfamily [5]. Thus, it came as a surprise to find that *mPing *was actively transposing in an *indica *rice cell culture line that lacked the *Ping *element [20]. The most likely source of TPase was determined to be the closely related *Pong *element, which is present in multiple copies in all tested strains of *Oryza sativa*. Subsequent studies with transgenic *Arabidopsis *confirmed that either Ping or Pong proteins were able to mobilize *mPing *and that transposition required functional copies of ORF1 and TPase [9].

Heterologous assay systems in plants and human cell culture have provided clues to the function of ORF1 protein and the reason why two proteins are required for transposition [9,10]. For most class 2 elements, the TPase contains a conserved catalytic domain (DDE) and a DNA binding domain that recognizes and binds to the terminal inverted repeat (TIR) and/or subterminal regions [23]. In contrast, the TPase of characterized members of the *PIF/Harbinger *superfamily lacks an obvious binding domain. Instead, a conserved Myb-like domain in ORF1 protein was hypothesized to be involved in DNA binding [5,6]. This model was supported by studies with *Harbinger3N_DR*, an artificial element whose reconstruction was guided by building consensus sequences from the zebrafish genome [10]. *Harbinger3N_DR *was mobilized in human cells only when both the reconstructed *Harbinger3_DR *TPase and ORF1 proteins were co-expressed [10]. Furthermore, ORF1 protein was shown to bind the *Harbinger3N_DR *TIRs and interact with TPase. Finally, this study found that interaction with ORF1 protein was required for nuclear localization of TPase [10]. These results suggest that ORF1 protein plays a critical role by positioning the TPase both in the nucleus and at the TIR where excision occurs.

We were motivated to develop a more facile assay system as a first step in the understanding of the amplification of MITEs and to dissect the complex transposition mechanism catalyzed by the *PIF*/*Harbinger *proteins. Here we report a yeast assay that recapitulates all of the features of excision and reinsertion of *mPing *as seen in plant systems. Furthermore, we demonstrate the validity of the yeast model by showing that a mutation of a putative nuclear export signal (NES) in the TPase increased transposition both in yeast and plants. These results provide a platform for further analysis of the regulation of other *PIF/Harbinger *elements and their relationships to *Tourist*-*like *MITEs.

## Results

### *mPing *transposition in yeast

**Figure 1:**
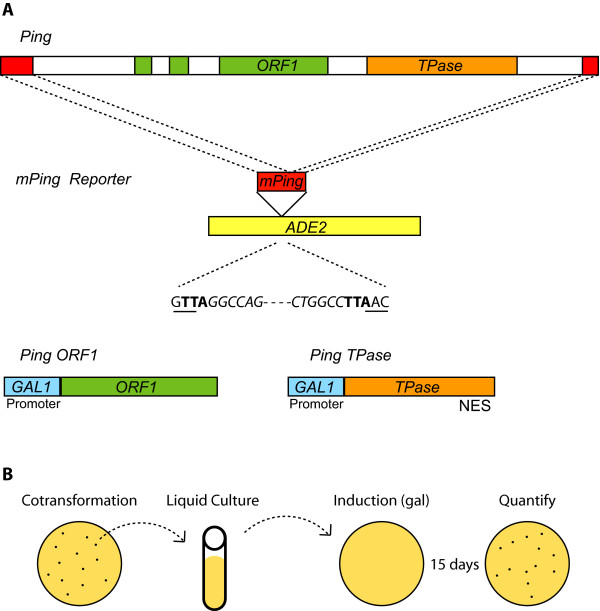
**Constructs used in this study and steps in the yeast assay**. Diagram showing the relationship of the native *Ping *and *mPing *elements to the constructs used for the yeast transposition assay (A). ORF1 (green) and TPase (orange) coding regions were placed in galactose inducible constructs, while *mPing *(red) was cloned into the *ADE2 *coding region (yellow). The transposition assay protocol (B) involves growing transformed yeast in non-inducing liquid media and then plating on inducing (galactose) media under selection for adenine autotrophy. *mPing *excision and repair of *ADE2 *results in colony formation.

In order to establish a transposition assay for *mPing *in yeast, we modified successful assays previously used for the maize *Ac*/*Ds *elements (the *hAT *superfamily) [24,25] and the rice *Osmar *elements (the *Mariner *superfamily) [9]. The most significant modification involved the construction of separate expression vectors containing fusions of the inducible yeast *GAL*1 promoter to either the *Ping *ORF1 or the *Ping *TPase coding regions (Figure 1). The third plasmid vector in this assay was a selectable marker composed of the yeast *ADE2 *gene with its coding region interrupted by *mPing *[including the TSD (TAA)] (Figure 1). Growth on medium lacking adenine requires the excision of *mPing *and the in-frame repair of *ADE2*. Expression of the Ping proteins (induced by galactose) and selection for excision was performed concurrently on solid media lacking adenine. In this assay, each colony represents a unique excision event, thus eliminating an artifactually high frequency due to founder effects from early transposition events. This strict selection is sensitive to slight changes in the health of the culture, due to factors such as age, density and growth phase. In recognition of this source of variation, we pooled the excision frequencies from two or more separate experiments, each with multiple replicates (see Additional File 1).

**Figure 2:**
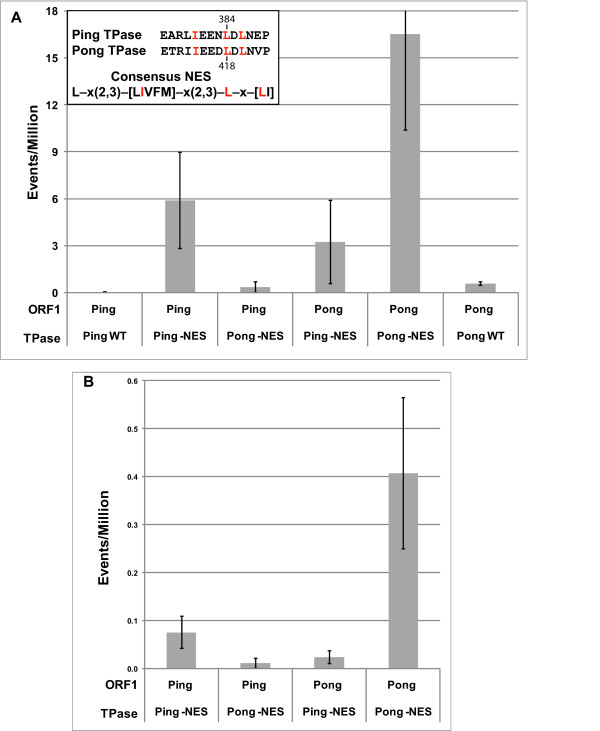
**Excision frequency in yeast catalyzed by wild type and mutant Ping and Pong proteins**. Histograms showing average excision frequencies for *mPing *(A) and *mPong *(B) in yeast using different combinations of Ping and Pong proteins. TPase - nuclear export signal (NES) mutants contain alanine substitutions at the two conserved leucines in the predicted NES (Ping 384+386, Pong 418+420). Error bars represent the standard error. Sequence of the NES for the Ping and Pong TPase proteins are shown in the top left corner. Residues that match the consensus NES are in red.

Co-expression of Ping TPase and ORF1 proteins in a single yeast cell resulted in a very low excision frequency of *mPing *from *ADE2 *(0.03 ± 0.02) (Figure 2, Additional File 1). We hypothesized that the low frequency could be due to the activity of a potential NES located in the C-terminal region of the TPase (Figure 2, Additional File 2) [26]. In order to determine the impact of this putative NES on activity, we mutated two leucine residues that are conserved in most predicted NESs (L384A, L386A)[26]. Alteration of this sequence resulted in significantly higher excision frequencies (5.9 ± 3) (Figure 2A, Additional File 1), suggesting that the NES was inhibiting TPase function in yeast. No *ADE2 *revertant colonies were observed when Ping ORF1 or TPase -NES proteins were expressed in isolation (data not shown).

The increased activity of the NES mutant provided the experimental resolution necessary to test the impact of site-directed mutations in conserved residues of domains predicted to be necessary for transposition. To test the suspected DNA binding domain of ORF1 protein, we disrupted the Myb-like sequence with a mutation in the helix-turn-helix fold (L225P). Co-expression of ORF1 (L225P) and the TPase -NES proteins resulted in no detectible excision. Similarly, mutation of the catalytic DDE domain of the TPase protein (D295A) led to a reduction in excision frequency of ~150-fold (0.04 ± 0.02) (Additional File 1). These results are consistent with the hypothesis that the DNA binding and catalytic functions are divided between the ORF1 and TPase proteins respectively.

### Cross-mobilization mediated by *Ping *and *Pong*

In rice and transgenic *Arabidopsis*, both Ping and Pong products can mobilize *mPing *even though their ORF1 and TPase proteins share only 62% and 77% amino acid identity, respectively [9,20]. To test whether Pong proteins can catalyze *mPing *transposition in yeast, we replaced Ping ORF1 and TPase with their Pong counterparts and combined these in yeast strains with the *mPing *reporter (see Methods). The results (Figure 2, Additional File 1) indicate that: (i) Pong proteins catalyze low frequency excision of *mPing; *(ii) introduction of the NES mutation (L418A, L420A) into the Pong TPase significantly increases the excision frequency; and (iii) Pong ORF1 functions with Ping TPase -NES and Ping ORF1 functions with Pong TPase -NES to catalyze *mPing *excision.

Another way to address the question of cross-mobilization is to ask whether Ping proteins can mobilize a deletion derivative of *Pong*. To this end, we generated a 500 bp nonautonomous *Pong *(*mPong*) and inserted it into the exact same position in *ADE2 *as *mPing *(see Methods). As shown in Figure 2B, while *mPong *is mobilized by the Ping and Pong proteins, the frequency is much lower than *mPing *(note the different scales on the Y axes). The possible reasons underlying this difference is revisited in the discussion.

### Analyses of excision and reinsertion events

**Figure 3:**
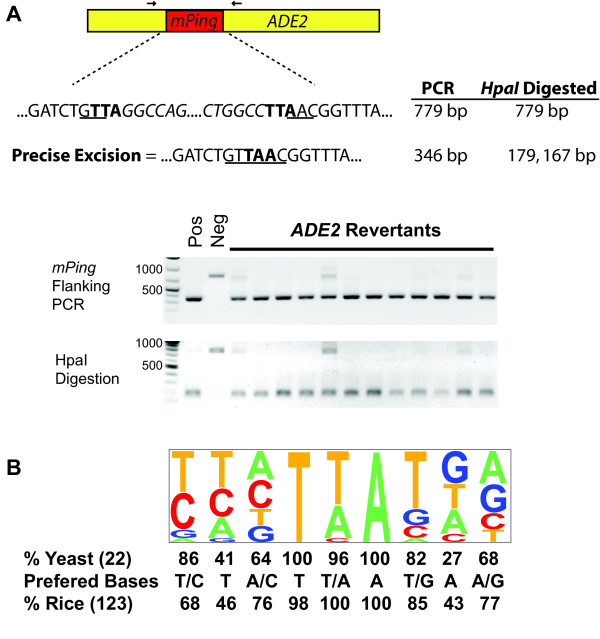
**Analysis of excision and insertion events**. Analysis of the *mPing *excision sites from *ADE2 *revertants by polymerase chain reaction (PCR) using flanking primers and digestion with *HpaI *(A). *mPing *excision results in smaller products that can only be digested with *HpaI *when the *ADE2 *gene is repaired perfectly. Some *ADE2 *revertant colonies carry both the original and excised versions of the reporter plasmids, resulting in the two PCR products shown. The *HpaI *site is underlined, the target site duplication (TTA) is shown in bold, and *mPing *sequence is italicized. (Neg = *mPing *reporter, Pos = *ADE2*) The *mPing *insertion preference in yeast is shown as a pictogram (height of letter indicates % at that position) and the frequencies of preferred nucleotides compared to rice [12] (B).

The high frequency of transposition catalyzed by the NES mutation permitted the isolation of many excision and reinsertion sites. Excision of *mPing *in both rice and transgenic *Arabidopsis *is unusual for a plant element in that it is usually precise [9,27]. In other words, there is no alteration (footprint) at the excision site because *mPing *and one copy of the 3 bp TSD (usually TAA) are almost always removed. In our yeast assay, precise excision could be easily detected because it restores the *Hpa*I restriction site used for cloning *mPing *into *ADE2 *(see Methods). We isolated yeast *ADE2 *revertants [*mPing *mobilized by Ping ORF1 and Ping TPase (L384/386A)] and analysed the excision sites by polymerase chain reaction (PCR) and *Hpa*I digestion (Figure 3A). Almost all of the excision events (109/110) were precise. The one imperfect event was due to a 3 bp deletion that maintained the reading frame of *ADE2 *(data not shown).

Another unusual feature of all characterized members of the *PIF/Harbinger *superfamily is an insertion sequence preference that extends beyond the 3 bp TSD. This was first observed for the maize *PIF *element, with a 9 bp extended insertion site preference (the TSD and 3 bp on each side) [4]. *mPing *was similarly shown to have a 9 bp insertion preference both in rice [12] and in transgenic *Arabidopsis *[9]. To investigate whether a preference was manifest in the yeast assay, we used inverse PCR and transposon display to isolate *mPing *reinsertion sites in the yeast genome (see Methods). A consensus sequence generated from the sequences of 22 independent events revealed a 9 bp insertion site preference that was similar to the *mPing *insertion preference in rice and transgenic *Arabidopsis *(Figure 3B, Additional File 3). The insertion of *mPong *elements was also verified by flanking sequence analysis for four events mobilized by Ping proteins and five events catalyzed by Pong proteins (Additional File 3).

### Protein localization

Our finding that the TPase NES had significant effects on activity in yeast suggested that protein localization is critical for *mPing *transposition. Many other TPase proteins harbor nuclear localization signals (NLS) that facilitates their access to the DNA substrate [28,29]. Analysis of Ping proteins with the nuclear prediction software NucPred indicated that the ORF1 protein has a potential NLS and, as such, is likely to be nuclear-localized (score = 0.9) [30]. In contrast, the TPase has a much lower NucPred score (0.37), indicating it may not utilize a NLS to gain access to the DNA.

**Figure 4:**
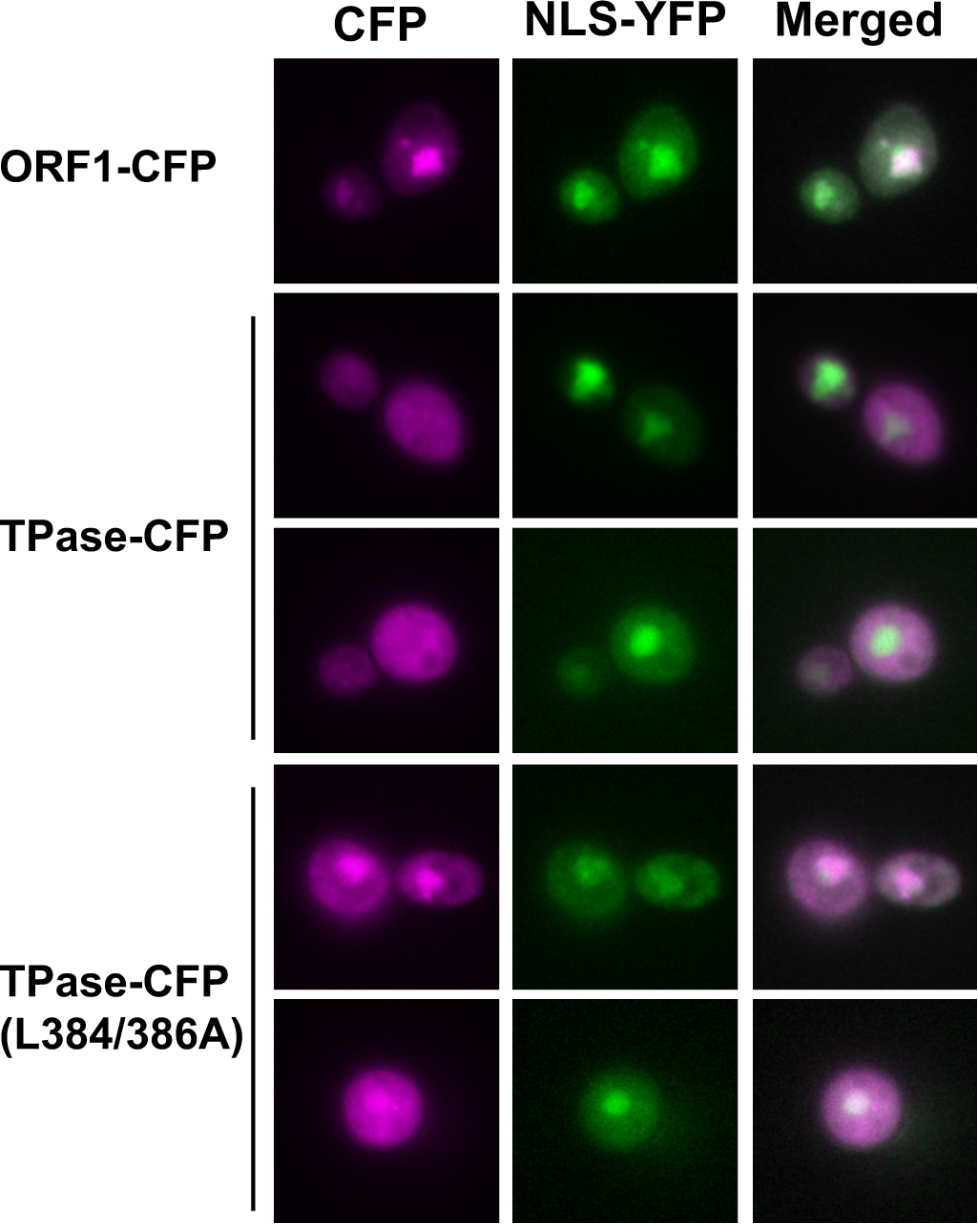
**Protein localization**. Fluorescence microscopy of yeast expressing cerulean fluorescent protein fusions of Ping ORF1, TPase or TPase (L384/386A) proteins (magenta). The nuclear localization signal-yellow fluorescent protein (green) was co-expressed to provide a nuclear marker.

We used cerulean florescent protein (CFP) fusions to test the localization of the Ping proteins in yeast. ORF1 protein was found to be primarily nuclear and co-localized with the nuclear marker protein, NLS-EYFP (Figure 4) [31]. In contrast, wild type TPase showed a diffuse staining pattern. Figure 4 shows that the TPase (L384/386A) NES mutant shows increased CFP fluorescence in the nucleus, confirming that the NES plays a role in nuclear export of the TPase. Co-expression of the Ping ORF1 and TPase proteins together did not significantly alter the localization pattern of either protein (data not shown).

### Analysis of the NES mutant in transgenic *Arabidopsis*

**Figure 5:**
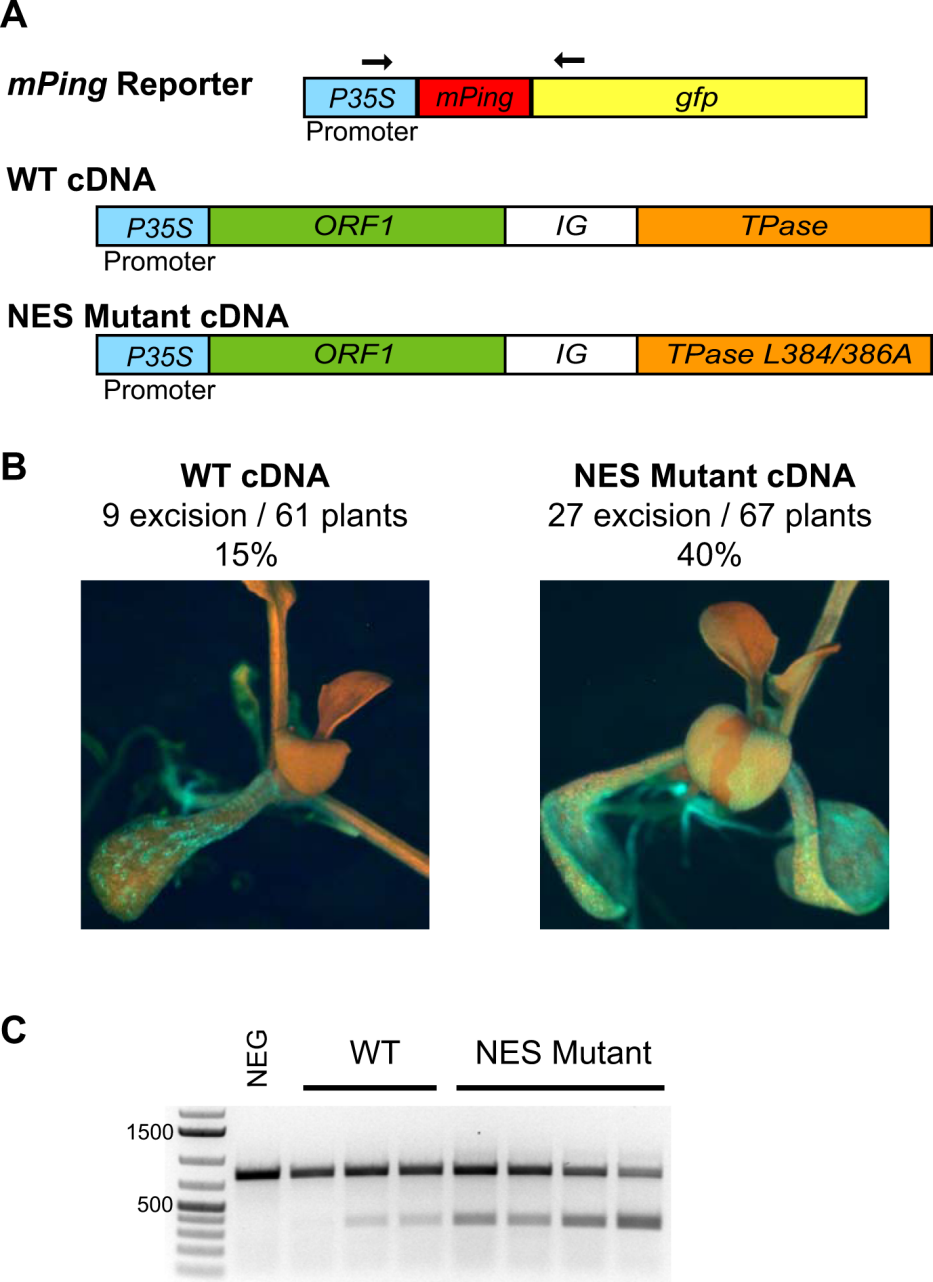
***Arabidopsis *transposition assay**. Comparison of the transposition activity of wild type TPase and the TPase (L384/386A) nuclear export signal mutant in *Arabidopsis *containing an *mPing gfp *reporter construct. ORF1 and TPase proteins were expressed from a cDNA that contains both genes [TPase is presumably expressed by a native promoter within the intergenic region] (A). The relative frequency of *mPing *excision for each construct is shown together with a representative green fluorescent protein (GFP) expressing plant (B). GFP expression produces green sectors on a red chlorophyll background. Polymerase chain reaction analysis of representative GFP expressing plants from each treatment using primers (shown in A) that flank the excision site (C).

Our findings indicate that the NES encoded by wild type TPase inhibits *mPing *transposition in yeast and that mutation of this motif increases nuclear localization and transposition activity. In order to determine if the NES also reduced transposition in plants, we modified the previously developed *Arabidopsis *transposition assay to test the effect of the TPase (L384/386) NES mutation. The reporter construct for this assay was *mPing *inserted between the CaMV 35S promoter and a *gfp *reporter gene (Figure 5A). We transformed the CaMV 35S:*Ping *cDNA with and without the TPase NES mutation (L384/386A) because the wild type construct was shown previously to express both ORF1 and TPase proteins from a single construct in *Arabidopsis *(see Methods) [9]. A comparison of the number of transgenic plants that showed *mPing *excision (determined by green fluorescent protein (GFP) visualization and PCR analysis) indicated that the alteration of the NES resulted in significantly higher excision activity (40% versus 15%, *P *< 0.005). In addition, the NES mutation resulted in more GFP fluorescence in both the cotyledons and mature leaves (Figure 5B). Finally, PCR analysis using primers that flank the *mPing *insertion site showed an increase in the amount of the ~400 bp (excision) product in seedlings containing the mutant TPase.

## Discussion

In this study we report the first yeast transposition assay for the *PIF*/*Harbinger *superfamily. The assay retains many of the unusual features of this superfamily including precise excision, insertion sequence preference and the requirement for the two element-encoded proteins. In order to develop this assay we used the related rice elements *Ping *and *Pong*, the only naturally active members of this superfamily, and *mPing*, the only naturally active *Tourist *MITE. Below, we discuss some of the features of this assay and how the wealth of yeast genetics could be exploited to understand the transposition mechanism and, perhaps, the success of *Tourist *MITEs.

### Excision and insertion

Excision of *mPing *in both rice and transgenic *Arabidopsis *is usually perfect as it involves the removal of both *mPing *and one copy of the TSD. This stands in marked contrast to all other plant class 2 elements where one or more nucleotides usually remain at the excision site as a so-called transposon footprint [11]. While all but one of the excision events of *mPing *in yeast were perfect (109/110) (Figure 3), one could argue that this result is an artifact of selection for *ADE2 *reversion. This would be the case if only the wild type sequence at this site was able to restore *ADE2 *function. However, in a parallel study of a rice *Stowaway *element, inserted at exactly the same site in *ADE2 *as *mPing*, all *ADE2 *revertants had footprints with insertions of 6, 9 or-12 bp [32].

Thus, excision site repair in yeast is influenced by currently unknown differences in the catalytic properties of the transposase source. Plant transposable element excision sites are thought to be repaired primarily by the non-homologous end-joining pathway (NHEJ), which is well characterized in yeast [33]. In a prior study, *Yu et al*. [25] analysed the excision of the maize *Ds *element by the *Ac *transposase (a member of the hAT superfamily) in yeast strains that were mutant for various steps in the NHEJ pathway. They found that mutations in a subset of the genes tested influenced both the quality and frequency of excision site repair [25]. In this regard, we have begun to analyse excision of *mPing *and the rice *mariner *element *OsStow35 *[32] in the same yeast mutant backgrounds to address why *mPing *but not *Ds *or *OsStow *is repaired perfectly.

Another distinguishing feature of the transposition reaction of characterized *PIF*/*Harbinger *elements is an extended insertion sequence preference of 9-15 bp [4-6]. That the target preference is conserved (to the extent that our yeast sample size can differentiate, Figure 3) indicates that one or both of the element-encoded proteins is responsible for this specificity. As such, the yeast assay should be a valuable tool that could allow the rapid analysis of mutant ORF1 and/or TPase proteins in order to determine if they contribute to insertion preference.

### Requirement for 2 element-encoded proteins

Perhaps the most unusual feature of the *PIF*/*Harbinger *superfamily is that transposition requires two element-encoded proteins, ORF1 and TPase [9,10]. Prior analysis of the reconstructed *Harbinger3N_DR *element from zebrafish suggested that ORF1 protein binds to the TIR and is required in human cells for nuclear localization of TPase (which lacks a nuclear localization sequence). In this study, the use of the wild type ORF1 and TPase proteins from either *Ping *or *Pong *resulted in negligible excision of *mPing *(Figure 2). This result was unexpected as these proteins catalyzed robust excision of *mPing *from the *gfp *reporter in transgenic *Arabidopsis *[9]. Mutagenesis of a predicted NES in the C-terminal region of the TPase from both *Ping *and *Pong *(Additional File 2) led to significant increases in the excision frequency of *mPing *(Figure 2A).

Increased nuclear localization of the mutant versus the wild type Ping TPase (Figure 4) suggests that the low excision frequency catalyzed by wild type TPase is a direct result of its export from the nucleus. However, if this is the case, why does wild type Ping and Pong TPase catalyze *mPing *excision in both rice and *Arabidopsis *[9,20]? One reason may be that yeast, like other simple eukaryotes, undergoes a 'closed' mitosis where the nuclear envelope does not break down [34]. Unlike the situation in plants, TPase exported to the cytoplasm in yeast cannot get back into the nucleus during mitosis. Thus, mutation of the NES prevents or slows nuclear export of TPase and keeps it in the nucleus.

An alternative model to explain the difference in activity of the wild type TPase in yeast and plants is that the NES may not function in rice and *Arabidopsis*. To address this question, we generated transgenic *Arabidopsis *plants with wild type and mutant TPase (Figure 5A). Although excision frequency is difficult to quantify precisely, we found that both the percentage of plants showing excision events and PCR products resulting from *mPing *excision were significantly increased in plants harbouring the TPase with the mutant NES (Figure 5). The identification of a hyperactive mutation in yeast, and its subsequent validation in plants, indicates the potential value of the yeast assay as a rapid and facile screen for ORF1 and TPase mutations that reduce or increase the frequency of transposition or alter the quality of excision or insertion events.

### Using the yeast assay to dissect the success of *mPing*

In addition to its potential utility at dissecting the catalytic features of the *PIF*/*Harbinger *superfamily, the yeast assay may be of value in determining the reason why *mPing*, and not other nonautonomous elements, become MITEs. As is true for many relatively recent discoveries, the definition of just what is a MITE is continuously being refined as an increasing number of genome sequences become available. Stated simply, a MITE is a class 2 nonautonomous element that can attain very high copy numbers (hundreds or thousands) through the rapid amplification of one or a few identical or nearly identical founder elements [35,36]. MITEs are usually short (~100-500 bp) and are either of unknown origin or, like *mPing*, are deletion derivatives of a larger autonomous element.

One of the outstanding questions about MITEs is whether their ability to attain high copy numbers is an inherent feature of the element or a fortuitous circumstance. In other words, is there something special about the structure of MITEs that, for example, makes them better substrates for transposase? Alternatively, are MITEs just average nonautonomous elements that happen to be in the genome when, for example, an unrelated autonomous element is producing transposase? The results of this study favour the former model where MITEs, such as *mPing*, have distinctive structural features (compared to other low copy nonautonomous elements) that facilitate their rapid amplification and spread in the genome. Although *mPing *and *mPong *are both deletion derivatives of larger autonomous elements, the excision frequency of the former is much higher than the latter (Figure 2A, B). In fact, the frequency of *mPing *excision is ~40- to 50-fold higher than that of *mPong *when either Ping or Pong proteins catalyze transposition. The relative success of *mPing *in yeast mirrors what occurs in rice; there are less than five deletion derivatives of *mPong *in all of the sequenced rice genomes, while most characterized strains of *japonica *rice have 25-50 *mPing *copies and a few have over 1000 [12]. As such, the yeast assay can be used to determine the excision frequencies of a variety of constructs, including additional deletion derivatives of *Pong *and *Ping*, chimeric elements where regions of *mPing *and *mPong *are swapped and site-directed mutations throughout *mPing*. In this way we should be able to determine the cis-features that distinguish MITEs from low copy number nonautonomous elements.

## Conclusions

This is the first report of the transposition of members of the *PIF/Harbinger *superfamily in yeast. This breakthrough facilitated the characterization of the NES encoded by the Ping and Pong TPase proteins and confirms a role for the control of nuclear access in regulating transposition. The yeast assay also provides a platform for the analysis of the unique characteristics of the transposition reaction including precise excision and insertion sequence preference. Our finding that Ping and Pong proteins can function in a cooperative manner suggests that a new layer of cross-mobilization may have to be considered in future studies of how transposable element families evolve. Finally, the transposition of *mPing *in yeast will facilitate investigations into the features underlying the success of *Tourist*-like MITEs.

## Methods

### Yeast strains and construct construction

*Saccharomyces cerevisiae *strain DG2523 and the pWL89a vector were described previously [24,37]. The following primers were used to prepare the elements for ligation into the *HpaI *site of *ADE2*: *mPing *F 5'-AAGGCCAGTCACAATGGGG-3', *mPing *R 5'-AGGCCAGTCACAATGGCTAG-3'; *mPong *F 5'-AAGGCCAGTCACAATGGGG-3'; and *mPong *R 5'-AGGCCATTCACAATGCAGT-3'. The *mPong *element was constructed in order to contain 251 bp from the 5' end of *Pong *and 245 bp from the 3' end of *Pong *(sequence available upon request). Genes from cDNA clones [GenBank: AK068363.1, GenBank: AK068654.1] or genomic DNA were cloned into the Gateway cloning vectors, pENTR or pDONR Zeo, and then transferred to destination vectors with LR Clonase reactions (Invitrogen, California, USA). Sequence alterations were made using the Quikchange II Mutagenesis Kit (Stratagene, California, USA).

### Yeast transposition assay

*Ping *and *Pong *ORFs (wild type and mutant) were cloned into pAG413GAL-ccdb (*ORF1*) and pAG415GAL-ccdb (*TPase*) vectors [38]. Transformed yeast were grown to saturation (36-48 h) in 5 ml of CSM -his-leu-ura with dextrose, washed with 5 ml sterile water, resuspended in 0.5 ml water and plated onto CSM -ade -his -leu -ura with galactose as the sole carbon source. Colonies were counted after incubation at 30°C for 15 days. Viable counts were made by plating 50 μl or 100 μl of a 4 × 10^4 ^dilution on yeast extract peptone dextrose plates.

### Excision and insertion site analysis

*ADE2 *revertant yeast was grown in selective media and genomic DNA isolations were performed using an E.Z.N.A. Yeast DNA Kit (Omega Bio-tek, Georgia, USA). PCR [PCR Master Mix (Promega, Wisconsin, USA)] using primers flanking the *mPing *excision site were digested with *HpaI *(New England Biolabs, Massachusetts, USA) and then analysed by gel electrophoresis. PCR products were cloned for sequencing using Topo-pCR2.1 (Invitrogen). Inverse PCR and transposon display was performed as described [9]. The insertion site analysis figure was made using the program Pictogram http://genes.mit.edu/pictogram.html.

### Microscopy

The yeast nuclear marker vector NLS-EYFP (pPS1888) was described previously [31]. N-terminal cerulean fusion protein constructs were constructed using pAG415GAL-cerulean-ccdb [38]. Yeast were grown on selective media with galactose, visualized with a Zeiss Axio Imager M1 microscope equipped with a 63× oil objective and SlideBook 4.0 software (Intelligent Imaging Innovations, Denver, USA). GFP expression was detected in seedlings with a Zeiss Discovery V12 fluorescence stereoscope.

### *Arabidopsis *transposition assay

The coding regions of the *Ping *cDNA [GenBank: AK068363.1] were cloned into the pENTR vector for mutagenesis and sequencing before transfer to the pEarleyGate 204 vector [39]. Wild type and NES mutant versions of TPase were transformed into *Arabidopsis thaliana *(Columbia ecotype) containing a previously transformed single copy of the *mPing *reporter [9] using a simple *Agrobacterium tumefaciens *(GV3103) floral dip method [40]. Seedlings were germinated on 0.2% phytagel plates (0.5 Murashige and Skoog, 1% sucrose) containing the appropriate selection reagents (7.5 μg/ml PPT, 50 μg/ml Kan, 150 μg/ml Timentin) in a Percival growth chamber (12 hrs light, 21°C) and analysed by fluorescent microscopy. DNA from 2-week-old seedlings was extracted by the CTAB method with PCR performed as described to confirm transformation and detect *mPing *excision [9].

## Abbreviations

CFP: cerulean fluorescent protein; GFP: green florescent protein; MITE: miniature inverted repeat transposable element; NES: nuclear export signal; NHEJ: non-homologous end joining; NLS: nuclear localization signal; PCR: polymerase chain reaction; TIR: terminal inverted repeat; TSD: target site duplication; YFP: yellow fluorescent protein.

## Competing interests

The authors declare that they have no competing interests.

## Authors' contributions

CNH designed and carried out the *Arabidopsis *transposition assays, microscopy and coordinated/participated in all of the yeast transposition assays and the associated sequence analysis. FZ participated in the design of the study, produced some yeast constructs and performed a portion of the insertion site analysis. SRW conceived the study, participated in its design and coordination and helped to draft the manuscript. All authors read and approved the final manuscript.

## Supplementary Material

Additional file 1 **Table of yeast excision frequency data**. Excel file containing the data set used to produce Figure 2. Frequency = *ADE2 *revertants/total cells plated. Click here for file

Additional file 2 **NetNES output**. PDF file containing the prediction of the nuclear export signal (NES) signal in the C-terminal region of the Ping TPase protein. NN indicates the neural network score, HMM indicates the hidden Markov model score. Scores above the threshold indicate potential NES signals. Click here for file

Additional file 3 **Insertion site sequences**. Excel file containing a table of the *mPing *and *mPong *insertions in yeast. Click here for file
