# Supplementary figures and images for: Transposition of the Tourist-MITE mPing in yeast: an assay that retains key features of catalysis by the class 2 PIF/Harbinger superfamily
Source: Mob DNA. 2010 Feb 1;1:5. doi: 10.1186/1759-8753-1-5 (PMC2836001; doi:10.1186/1759-8753-1-5)

NetNES 1.1: Predicted NES signals in AK068363

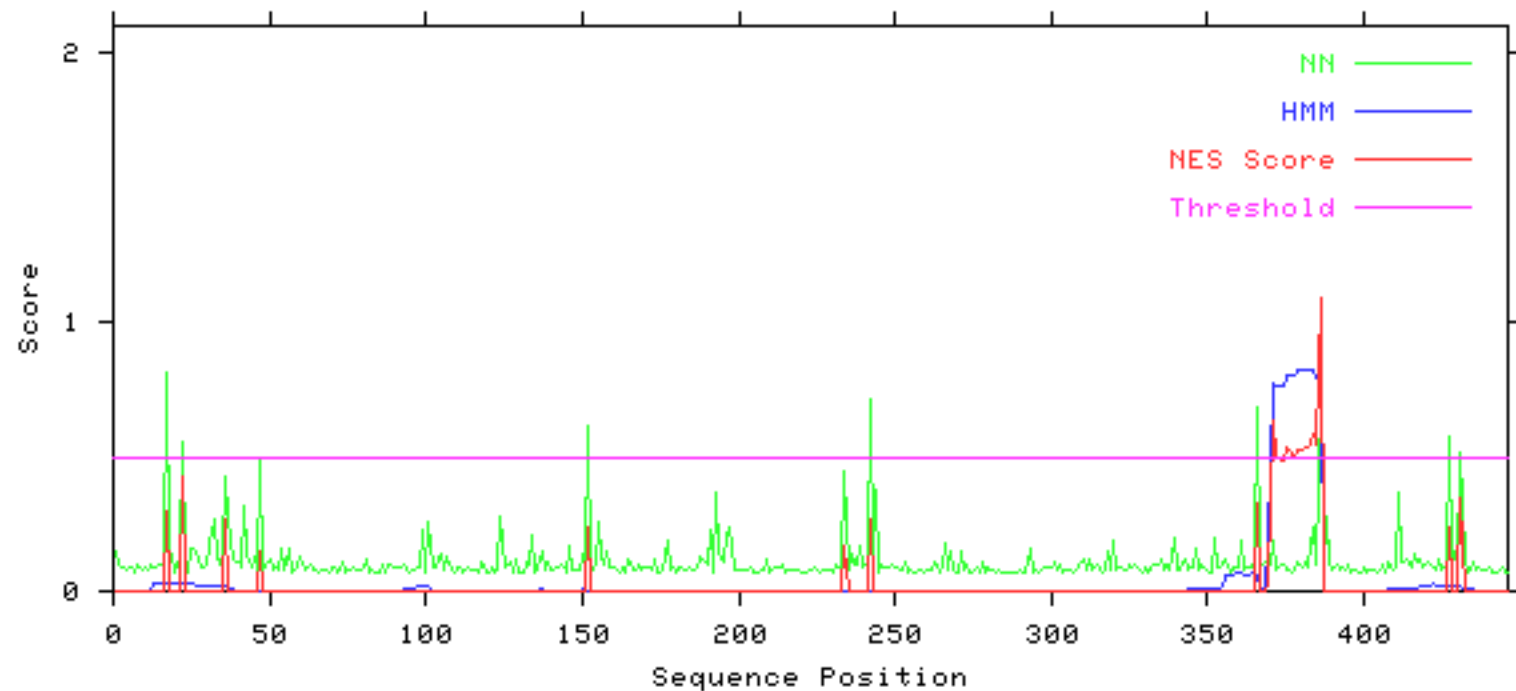

Supplement: Additional file 2 — NetNES output. PDF file containing the prediction of the nuclear export signal (NES) signal in the C-terminal region of the Ping TPase protein. NN indicates the neural network score, HMM indicates the hidden Markov model score. Scores above the threshold indicate potential NES signals. [file 1759-8753-1-5-S2.PDF]
